# Large orbital polarization in nickelate-cuprate heterostructures by dimensional control of oxygen coordination

**DOI:** 10.1038/s41467-019-08472-y

**Published:** 2019-02-04

**Authors:** Zhaoliang Liao, Elizabeth Skoropata, J. W. Freeland, Er-Jia Guo, Ryan Desautels, Xiang Gao, Changhee Sohn, Ankur Rastogi, T. Zac Ward, Tao Zou, Timothy Charlton, Michael R. Fitzsimmons, Ho Nyung Lee

**Affiliations:** 1 Materials Science and Technology Division, Oak Ridge National Laboratory, Oak Ridge, TN 37831 United States; 2 Advanced Photon Source, Argonne National Laboratory, Argonne, IL 60439 United States; 3 Neutron Scattering Division, Oak Ridge National Laboratory, Oak Ridge, TN 37831 United States; 4 Department of Physics and Astronomy, University of Tennessee, Knoxville, TN 37996 United States

## Abstract

Artificial heterostructures composed of dissimilar transition metal oxides provide unprecedented opportunities to create remarkable physical phenomena. Here, we report a means to deliberately control the orbital polarization in LaNiO_3_ (LNO) through interfacing with SrCuO_2_ (SCO), which has an infinite-layer structure for CuO_2_. Dimensional control of SCO results in a planar-type (P–SCO) to chain-type (C–SCO) structure transition depending on the SCO thickness. This transition is exploited to induce either a NiO_5_ pyramidal or a NiO_6_ octahedral structure at the SCO/LNO interface. Consequently, a large change in the Ni *d* orbital occupation up to ~30% is achieved in P–SCO/LNO superlattices, whereas the Ni *e*_g_ orbital splitting is negligible in C–SCO/LNO superlattices. The engineered oxygen coordination triggers a metal-to-insulator transition in SCO/LNO superlattices. Our results demonstrate that interfacial oxygen coordination engineering provides an effective means to manipulate the orbital configuration and associated physical properties, paving a pathway towards the advancement of oxide electronics.

## Abstract (web summary)

In correlated materials, physical properties depend on orbital occupancy and polarization. Here, a way to control the oxygen coordination via dimensionality of superlattices is presented that results in the change of orbital occupancy by 30%, which is larger than what has been achieved by other methods.

## Introduction

The correlated *d*-orbital plays a central role in creating collective phenomena in transition metal oxides, since the *s*-electrons of transition metals are transferred to oxygen ions, and the remaining *d*-electrons determine the delicate interplay between spin, charge, and orbital degrees of freedom^1^. For example, superexchange can induce either antiferromagnetic or ferromagnetic coupling of spins depending on the orbital filling and ordering, yielding a vast array of magnetic ground states^2–5^. In high transition temperature (*T*_C_) cuprate superconductors, the *x*^*2*^ *−* *y*^*2*^ orbital character is crucial for Cooper pairing^6^. The remarkable Mott metal-insulator transition often found in correlated oxides can be engineered by tailoring the orbital occupancy^7^. Thus, identifying ways to deliberately control the orbital degree of freedom in correlated oxides is highly desirable to discover electronic and magnetic phenomena that are useful for developing oxide electronic and spintronic devices. Interface engineering is a powerful method to manipulate such orbital degrees of freedom^7–13^ and has revealed many intriguing phenomena not possible in bulk material counterparts, such as interfacial charge transfer, orbital covalency, and broken inversion symmetry at nanometer scale^12–19^.

Rare-earth nickelate (RNiO_3_) heterostructures provide good experimental and theoretical frameworks to investigate influence of orbital occupation on the physical properties^17–24^. The *d*^7^ ionic configuration of Ni^3+^ in nickelates contains a full *t*_2g_ shell and a single electron occupying the *e*_g_ orbital. This orbital configuration generally favors a Jahn–Teller distortion as observed in LaMnO_3_^25^. However, the *e*_g_ orbitals in nickelates are doubly degenerate. A recent theoretical study proposed that the planar *x*^*2*^*-y*^*2*^ orbital order could be realized by spatially confining LaNiO_3_ into a ultrathin layer between two insulators, leading to a cuprate-like Fermi surface and, subsequently, high *T*_C_ superconductivity^23,24^. This idea triggered enormous experimental efforts to study the RNiO_3_ systems. However the orbital polarization caused by spatial confinement and strain was much smaller than the predicted one^17,26–28^. Resonant inelastic X-ray scattering indicated a very minor *e*_g_ splitting energy $( {\Delta E_{e_{\mathrm{g}}}} )$ induced by heterostructural engineering^29^. It has also been suggested that nickelate thin films prefer to retain their preferred electronic and structural configurations determined by the bulk phase, which limits the control of the orbital parameters through heterostructural engineering^30^.

**Fig. 1:**
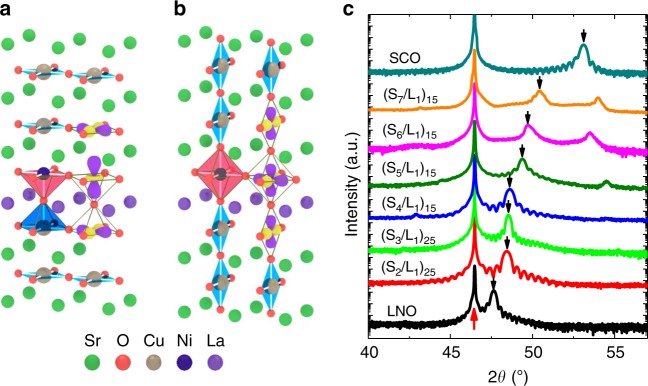
Interfacial engineering of oxygen coordination in SrCuO_2_/LaNiO_3_ superlattices. Schematics for SrCuO_2_/LaNiO_3_ (SCO/LNO) heterostructures with **a** planar-type SrCuO_2_ and **b** chain-type SrCuO_2_, resulting in different oxygen coordination and orbital polarization of Ni ions in LaNiO_3_. **c** X-ray diffraction (XRD) *θ*–2*θ* scans of a LaNiO_3_ film (34 nm in thickness), ((SrCuO_2_*)*_*N*_/(LaNiO_3_)_1_)_*M*_ (noted as (S_*N*_/L_1_)_*M*_) superlattices, and a SrCuO_2_ film (28 nm). The red and black arrows indicate the SrTiO_3_ 002 peak and the peaks from superlattices or films, respectively, showing a clear modulation of out-of-plane lattice constants

Here, we report that one can largely manipulate the occupancy of Ni 3*d* orbitals in LNO-SrCuO_2_ (SCO) heterostructures by dimensional control of the SCO layers. The SCO which in the bulk phase is orthorhombic (Cmcm) with a double-chain structure formed in a perovskite like framework with missing apical oxygen in thin films^31,32^. Upon reducing the thickness when grown in thin films, the planar-type structure of SCO (P–SCO) is transformed to a chain-type structure (C–SCO)^33–35^. The CuO_2_ planes of P–SCO and C–SCO are parallel and perpendicular with respect to the film plane, respectively. This difference effectively yields two different heterostructures (see Fig. 1a, b). In the case of a P–SCO/LNO heterostructure grown on a B-site terminated ABO_3_ perovskite substrate, the interfacial atomic stacking sequence is hypothesized to be (CuO_2_/Sr)/(NiO_2_/LaO), leading to a truncated NiO_5_ pyramid structure (see Fig. 1a). On the other hand, the stacking sequence for the C–SCO/LNO interface would become (CuO/SrO)/(NiO_2_/LaO), which yields an octahedrally coordinated Ni and thus no modification of the LNO structure (see Fig. 1b). Our results demonstrate how the unique infinite-layer structure offered in SCO can be utilized to induce a very large Ni 3*d* orbital polarization in P–SCO/LNO superlattices or to create degenerate (i.e., little or no polarization) Ni *e*_g_ orbitals in C–SCO/LNO superlattices as sketched in Fig. 1a, b. The observed changes in the electronic and orbital states may have a profound impact on transport properties of SCO/LNO superlattices.

## Results

### Dimensional control of the sub-lattice structure of SrCuO_2_

LNO and SCO films and their superlattices were grown on TiO_2_ terminated (001) SrTiO_3_ (STO) substrates by pulsed laser epitaxy. The growth condition was optimized to achieve high-quality SCO films in the bulk planar structure (see Supplementary Fig. 1), and all films were atomically flat with step-terrace features on their surfaces (See Supplementary Fig. 2). As shown in Fig. 1c, X-ray diffraction (XRD) *θ*–2*θ* scans of an 80 unit-cell (u.c.) SCO film revealed clearly the SCO 002 peak near 2*θ* = 53^o^, confirming the bulk-like infinite-layer structure^33,34^. Using the same growth conditions, high-quality LNO films were obtained as well. When growing (SCO_*N*_/LNO_1_)_*M*_ superlattices (noted as S_*N*_/L_1_), a systematic change in XRD spectra was observed with increasing the SCO layer thickness. Since the P–SCO and C–SCO have quite different out-of-plane lattice constants of ~3.45 Å (2*θ* = 53^o^) and ~3.8 Å (2*θ* = 47.8^o^), respectively^33,35^, the position of the main superlattice peak SL(0) can be used to identify the SCO structure type. For relatively thick SCO layers (e.g., *N* = 7 u.c.), SL(0) was located between the SCO 002 and the LNO 002 XRD peaks, suggesting the formation of the planar-type SCO structure within the superlattice. When the SCO layer thickness is reduced to 2 u.c., the SL(0) peak shifts close to the LNO 002 peak, indicating that 2 u.c.-thick SCO has a similar out-of-plane lattice constant with that of LNO. This result suggests that a chain-type SCO structure was formed in the S_2_/L_1_ superlattice. With increasing the SCO film thickness to 5 u.c., a sudden large shift of the superlattice peaks occurred. This shift suggests the critical thickness of the phase conversion from P–SCO to C–SCO to be at 5 u.c.. This thickness-driven transition of the SCO structure in SCO/LNO superlattices was also observed for other LNO sublayer thicknesses (see Supplementary Fig. 3).

**Fig. 2:**
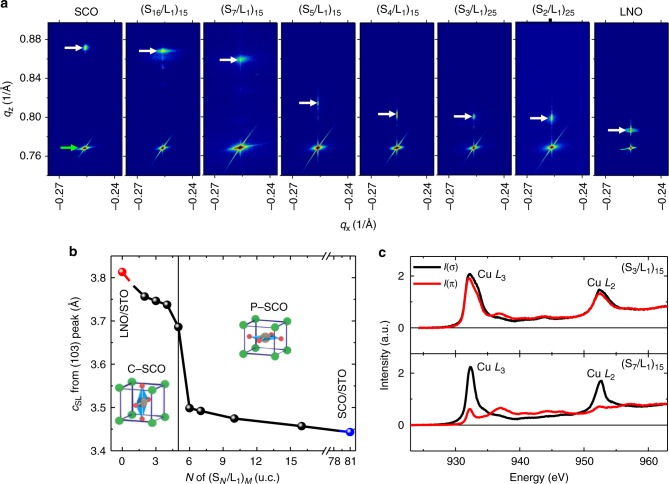
Dimensional control of the sub-lattice structure of SrCuO_2_. **a** Reciprocal space mapping of the 103 peak of a LaNiO_3_ (LNO) film, ((SrCuO_2_)_*N*_/LNO_1_)_*M*_ superlattices and a SrCuO_2_ (SCO) film. The green and white arrows indicate SrTiO_3_ (STO) substrate and film/superlattice peaks, respectively. **b** Out-of-plane lattice constant (*c*) as a function SCO sublayer thickness (*N*). The sudden change of *c* across *N* = 5 u.c. indicates a structural transition from a planar type to a chain type. **c** Polarization dependent XAS of Cu *L*_2,3_ edge for S_7_/L_1_ (top panel) and S_3_/L_1_ (bottom panel) superlattices, demonstrating a clear contrast on the orbital population

Further evidence of the structural transition triggered by reducing the SCO thickness was observed from XRD reciprocal space mapping (RSM). The RSM shown in Fig. 2a indicates that all the superlattices and reference SCO and LNO films were coherently grown on STO substrates. The reciprocal lattice vector (*q*_z_) of thick SCO superlattices (e.g., *N* = 7 and 16 in S_*N*_/L_1_) was very close to that of a P–SCO film, confirming that the SCO layers in the superlattices possess the bulk-like planar-type structure. On the other hand, the *q*_z_ of thin SCO superlattices (*N* = 2–4) was similar to that of a LNO film. Interestingly, as shown in Fig. 2b, the SCO thickness dependent change in the out-of-plane lattice constant (*c*_SL_) calculated from the RSMs around the 103 peak (*c*_SL_ = 3/*q*_z_) clearly reveals a sudden transition at around 5 u.c. of the SCO sublayer thickness. If the bulk-like P–SCO is maintained when the SCO thickness is reduced, one can only expect a gradual increase in *c*_SL_. Therefore, our observation of the step-like transition shown in Fig. 2a, b unambiguously demonstrates that the planar-type lattice structure of SCO can be converted into the chain-type structure by reducing the SCO thickness below 5 u.c. Detailed XRD and transport analyses further suggest the formation of a twin domain structure within the chain-type SCO (see supplementary Fig. 4), originating from the two-fold symmetry of C–SCO on the four-fold (001) STO substrate.

Such a change in the orientation of the oxygen plane can be confirmed as a change in the orbital occupancy measured from linearly polarized X-ray absorption spectroscopy (XAS) acquired in fluorescence yield (FY) mode. The orbital occupancy of Cu *x*^*2*^-*y*^*2*^ and *3z*^*2*^*-r*^*2*^ orbitals was probed by the XAS measured with in-plane (σ) and out-of-plane (π) polarized x-rays, respectively. As shown in Fig. 2c, the large *I*(σ) versus *I*(π) XAS of the Cu *L*_2,3_-edge of the S_7_/L_1_ superlattice indicates exclusive $3d_{x^2 - y^2}$ character, consistent with orbital configuration in layer-structured cuprates (see supplementary Fig. 5). By contrast, *I*(σ) and *I*(π) share a similar intensity and shape for the S_3_/L_1_ superlattice, suggesting the chain-type SCO structure and $3d_{y^2 - z^2}$orbital character^36^. Additionally, the change in the SCO microstructure and oxygen coordination of Ni were confirmed by scanning transmission electron microscopy (STEM) (see Supplementary Fig. 6), consistent with XRD and XAS data.

### Engineered Ni orbital polarization in SrCuO_2_-LaNiO_3_ superlattices

**Fig. 3:**
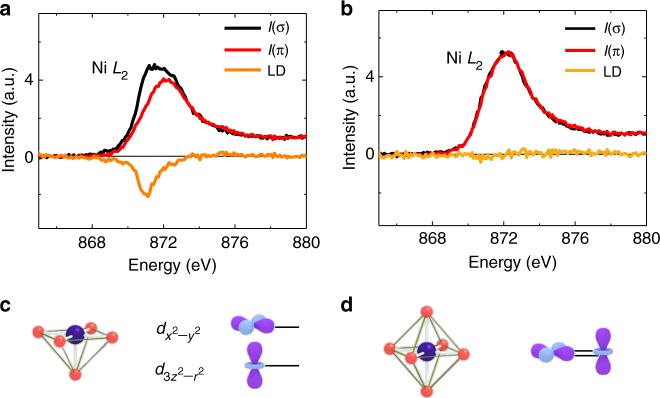
Orbital polarization of the Ni-3*d* orbital by oxygen coordination control. **a** Polarization dependent X-ray absorption spectroscopy (XAS) of Ni *L*_2_ edge for **a** S_7_/L_1_ and **b** S_3_/L_1_ superlattices. Large linear dichroism (LD) is observed for the S_7_/L_1_ SL, whereas the LD is nearly zero for the S_3_/L_1_ SL. Schematics of the *e*_g_ orbital configuration are shown for **c** S_7_/L_1_ and **d** S_3_/L_1_ superlattices

Now we turn to investigate the reconstruction of the Ni *e*_*g*_ orbital. Figure 3a shows the XAS of the Ni *L*_2_-edge of an S_7_/L_1_ superlattice. Since the Ni *L*_3_-edge largely overlaps with the La *M*-edge, and FY of the La^3+^ (4*f*^*0*^) *M*-edge deviates from the true XAS for the conventional detection geometry used, the Ni *L*_2_-edge is more suitable to illustrate the orbital reconstruction (see supplementary Fig. 7)^17^. The *I*(σ) exhibits a higher intensity than *I*(π), indicating that holes prefer to occupy the $3d_{x^2 - y^2}$orbital. For the S_3_/L_1_ superlattice, the *I*(σ) and *I*(π) intensities are similar to each other. Therefore, the Ni *e*_g_ orbital is degenerate (Fig. 3b). The different orbital configuration between the S_7_/L_1_ superlattice and the S_3_/L_1_ superlattice is also observed in total electron yield mode (see supplementary Fig. 7), indicating that the observed polarization dependence of the FY XAS originates from the intrinsic orbital polarization. The orbital configuration in the S_3_/L_1_ superlattice confirms the chain-type SCO structure within the S_3_/L_1_ superlattice, which recovers the octahedral coordination. Using the difference between in-plane polarized absorption *I*(*ab*) and out-of-plane polarized absorption *I*(*c*), the ratio of unoccupied states, *r*, can be quantified using the following sum rule^17,37^

1 $$r = \frac{{h_{3z^2 - r^2}}}{{h_{x^2 - y^2}}} = \frac{{3I(c)}}{{4I(ab) - I(c)}}$$

Here, *a*, *b*, and *c* are the Cartesian coordination axis of the substrates with *c* along out-of-plane direction and *h* is the hole occupancy. The π-polarized absorption *I*(π) was corrected for the angle *θ* = 20^o^ between the incident X-rays and sample plane in our experimental setup using *I*(*c*) = [*I*(π) − *I*(*ab*)sin^2^*θ*]/cos^2^*θ*, while the orientation of the incident beam with respect to the sample did not affect *I*(σ) and *I*(*ab*) = *I*(σ). The linear dichroism (LD) [*LD* = *I*(*c*) − *I*(*ab*)] shown in Fig. 3a, b further emphasizes the orbital polarization of the S_7_/L_1_ superlattice. Using the integrated intensity of *I*(*ab*) and *I*(*c*) from background-subtracted XAS, the hole ratio *r* can be quantified (see supplementary Fig. 8). The *r* values for S_7_/L_1_ and S_3_/L_1_ superlattices are 0.7 and 1.0, respectively, suggesting a predominant *x*^*2*^ *−* *y*^*2*^ hole character in S_7_/L_1_ and degenerate orbital in S_3_/L_1_. Previously, the *r* value of the largest orbital polarization $[ {P = ( {n_{x^2 - y^2} - n_{3z^2 - r^2}} )/( {n_{x^2 - y^2} + n_{3z^2 - r^2}} )} ]$ achieved by inducing tensile strain was ~1.19^26^. The spatial confinement in LAO/LNO superlattices has been reported to result in a value of *r* ~ 1.05^17,27^. The hole ratio that we observed cannot be compared directly to these previous values since our experiments had a flipping orbital configuration in which the $3d_{3z^2 - r^2}$orbital has more hole occupancy. The relative change of occupancy (≡|1 − *r*|) is instead used here for a quantitative comparison. In comparison to ~19% by strain^26^ and ~5% by spatial confinement^17,27^, the observed 30% change of Ni orbital occupancy in P–SCO/LNO superlattice confirms the significant impact of oxygen coordination on orbital reconstruction.

The prevailing $3d_{x^2 - y^2}$ character cannot be explained by spatial confinement, which favors the $3d_{3z^2 - r^2}$ hole occupancy due to the confinement of the $3d_{3z^2 - r^2}$ orbital in the *z* direction^17,23,24,38^. We attribute the observed large orbital polarization in the S_7_/L_1_ superlattice to the NiO_5_ pyramid structure, which is expected to lower the $3d_{3z^2 - r^2}$ with respect to $3d_{x^2 - y^2}$^39^. In fact, our polarization dependent XAS revealed quite similar features to the square-planar La_4_Ni_3_O_8_ and YBa_2_Cu_3_O_7_ (YBCO) single crystals^36,40^. The La_4_Ni_3_O_8_ is comprised of NiO_2_ planar structures while the main building block of YBCO is a CuO_5_ pyramid^40,41^. These two materials possess similar *e*_g_ band structures with the $3d_{3z^2 - r^2}$ lying lower than $3d_{x^2 - y^2}$. In sharp contrast, there is a negligible orbital polarization when the apical oxygen is retained in the S_3_/L_1_ superlattice. Although there is still tensile strain on LNO within the S_3_/L_1_ superlattice, the observed degenerate *e*_g_ orbital indicates that the octahedrally coordinated Ni prefers to retain a bulk-like electronic configuration. This retainment of bulk orbital configuration was reported previously^30^ and is also observed in our LNO films (see Supplementary Fig. 9). The corresponding *e*_g_ orbital energy diagrams for the NiO_5_ pyramid and the NiO_6_ octahedron are schematically plotted in Fig. 3c (S_7_/L_1_) and Fig. 3d (S_3_/L_1_). Using the outermost filled orbital, a sketch of the orbital configuration in SCO/LNO superlattice is plotted Fig. 1a, b, demonstrating the designed orbital configuration through heterostructural engineering.

### The charge states of Cu and Ni at interfaces

**Fig. 4:**
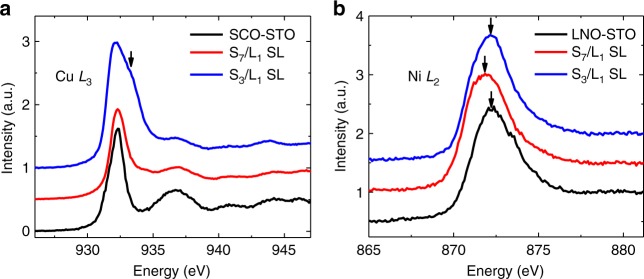
The charge states of Cu and Ni in different superlattices. **a** X-ray absorption spectroscopy (XAS) of Cu *L*_3_-edge for a SrCuO_2_ (SCO) film and (SCO_7_/(LaNiO_3_)_1_) (noted as S_7_/L_1_) and S_3_/L_1_ superlattices (SL). A shoulder indicated by the black arrow arises from Cu d^9^L state. **b** XAS of Ni *L*_2_-edge for a LaNiO_3_ (LNO) film and S_7_/L_1_ and S_3_/L_1_ superlattices. A slight shift of the peak position to a lower energy for S_7_/L_1_ superlattice suggests an electron doping in the LNO layer. The XAS is obtained by averaging linearly polarized XAS (*I* = (*I*(σ) + *I*(π))/2)

The charge states of Cu and Ni are further investigated using XAS. As shown in Fig. 4a, the Cu *L*-edge spectrum of a S_7_/L_1_ superlattice shows a characteristic Cu^2+^ feature as observed in SCO films^36,42^. Meanwhile, the S_3_/L_1_ superlattice shows a strong shoulder at ~933.28 eV, which is positioned ~1 eV higher than that of the Cu^2+^ peak and is consistent with the d^9^L state^36,42^. Here L represents the ligand hole. The doped holes are unlikely from interfacial charge transfer, since the Ni in S_3_/L_1_ maintains Ni^3+^ as demonstrated by the lack of Ni *L*-edge shift (see Fig. 4b). Further, La interdiffusion should favor Cu^1+^. It is more likely that the hole arises from oxygen excess SrCuO_2+*x*_. For the S_7_/L_1_ superlattice, the Ni *L*-edge shifts by about −0.3 eV relative to that of the LNO film. Since a 1 eV shift corresponds to roughly one electron valence change^43,44^, the energy shift suggests an averaged Ni^2.7+^ valence state in S_7_/L_1_. It has been reported that doped electrons at a LNO/La_2_CuO_4_ interface are predominantly accommodated by change of Ni valence^45^. A similar situation may occur for a SCO/LNO interface where a possible formation of oxygen vacancies may dope the interface state with electrons.

### Controlled metal-to-insulator transition

**Fig. 5:**
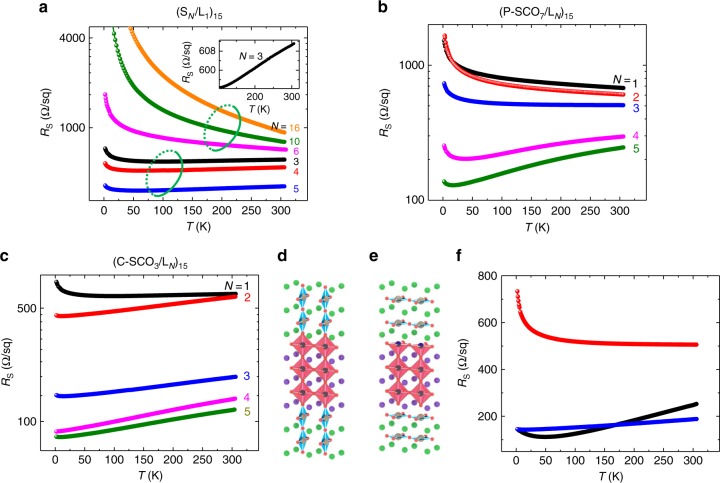
Metal-to-insulator transition by dimensionality control in superlattices. Temperature dependent sheet resistance of **a** (S_*N*_/L_1_)_15_, **b** (P–SCO_7_/L_*N*_)_15_, and **c** (C–SCO_3_/L_*N*_)_15_ for various layer thickness (*N*) of **a** SCO or **b**, **c** LNO. The green ellipses in **a** group separately the metallic and insulating superlattices. S, L, P–SCO, and C–SCO represent SrCuO_2_ (SCO), LaNiO_3_ (LNO), planar-type SCO, and chain-type SCO, respectively. Structural models for **d** (C–SCO_7_/L_3_)_15_ and **e** (P–SCO_3_/L_3_)_15_ superlattices. **f** Temperature dependent sheet resistance of (C–SCO_7_/L_3_)_15_ (blue curves), (P–SCO_3_/L_3_)_15_ (red curves), and (STO_7_/L_3_)_15_ (black curves). The transport properties illustrate a metal-to-insulator transition in 3 u.c. LNO layer when the top surface is transformed from the octahedral coordination to the pyramidal coordination

Functionally, we see that these observed changes to electronic and orbital states have a profound impact on transport properties of SCO/LNO superlattices. Figure 5a shows the transport properties of S_*N*_/L_1_ superlattices with different SCO thicknesses. The S_*N*_/L_1_ superlattices exhibit a metallic behavior (d*R*/d*T* > 0) to insulating behavior (d*R*/d*T* < 0) transition (MIT) with increasing the SCO layer thickness. The critical thickness for MIT is 5 u.c., which coincides with the critical thickness for a structural transition (see Fig. 2). This fact suggests that the P–SCO_*N*_/LNO_1_ and C–SCO_*N*_/LNO_1_ superlattices are insulating and metallic, respectively. Since LNO will undergo a MIT with reducing thickness across ~3–5 u.c.^46–49^, the single unit-cell LNO should be insulating and thus the metallic behavior arises from the C–SCO layer, which can be explained by the hole doping as observed by XAS (see Fig. 4a).

Figure 5b, c show the LNO layer thickness dependent transport properties in P–SCO_7_/L_*N*_ and C–SCO_3_/L_*N*_ superlattices, respectively. With reducing thickness of LNO layers, the P–SCO_7_/L_*N*_ superlattices gradually evolve from a metallic state to an insulating state. Such behavior is similar to MIT in single LNO films, which is associated with dimensional crossover^46,49^. What is more, the 3 u.c. critical thickness for MIT in P–SCO_7_/L_*N*_ is also similar to bare LNO films^46–49^. These results strongly suggest that the MIT in P–SCO_7_/L_*N*_ is driven by LNO sub-layers. Since the C–SCO is metallic, all C–SCO_3_/L_*N*_ superlattices exhibit good metallic behavior regardless of the thickness of LNO (see Fig. 5c). However, there is still a sharp decrease of the resistance when the LNO thickness is reduced from *N* = 3 to 2 u.c., while the C–SCO_3_/L_1_ and C–SCO_3_/L_2_ showed similar resistance values. This result implies a MIT in the LNO layers when reducing LNO thickness from 3 to 2 u.c. The different critical thickness (*t*_c_) for MIT of LNO in P–SCO_7_/L_*N*_ (*t*_c_ = 3) and in C–SCO_3_/L_*N*_ (*t*_c_ = 2) indicates that a MIT in 3 u.c. LNO sublayer occurs when the top surface of the LNO layer is transformed from the octahedral coordination to the pyramidal coordination (Fig. 5d, e). This result is further emphasized by comparing the resistance of P–SCO_7_/L_3_ with C–SCO_7_/L_3_ superlattices in Fig. 5f. Although the metallic C–SCO layer also contributes to the transport, the transport behavior of C–SCO_3_/L_3_ is dominated by the LNO layer since the resistance of C–SCO_3_/L_1_ is ~2 times larger than that of C–SCO_3_/L_3_. To further confirm the effect of oxygen coordination on transport, we measured the transport properties of STO_7_/L_*N*_ superlattices where the LNO interface has the same octahedral coordination as that in C–SCO_3_/L_3_. The critical thickness for MIT in STO_7_/L_*N*_ is 2 u.c. (see Supplementary Fig. 10), which is the same with C–SCO_3_/L_*N*_ superlattices and thinner than P–SCO_7_/L_*N*_ superlattices. This indicates that recovering the top pyramid NiO_5_ in P–SCO_7_/L_3_ to a full octahedral structure in STO_7_/L_3_ or C–SCO_3_/L_3_ will drive the LNO layer to be metallic. As shown in Fig. 5f, the STO_7_/L_3_ is metallic and has a similar resistance with C–SCO_3_/L_3_. The relative small change of resistance from P–SCO_7_/L_3_ to STO_7_/L_3_ is due to the crossover MIT in LNO and also the fact that P–SCO is not a good insulator and contributes to the transport as well. Higher LNO resistance in P–SCO/LNO superlattices could be due to the reduced oxygen coordination, as observed in bare LNO films^50,51^. The smaller Ni *e*_g_ bandwidth in pyramidal coordination and reduced in-plane electron hopping strength due to preferential 3*z*^2^-*r*^2^ orbital occupation would increase the resistance. Further, the appearance of Ni^2+^ may also play a role by opening a charge excitation gap^38,43^.

## Discussion

In conclusion, we have demonstrated that the orbital polarization and transport properties of SCO/LNO superlattices can be manipulated by templating the infinite structure of SCO into LNO through dimensionally controlling the oxygen coordination. Reducing the SCO thickness below 5 u.c. resulted in a structural transition from a planar-type to a chain-type structure. At SCO/LNO interfaces, the planar-type SCO structure induced a NiO_5_ pyramidal structure in LNO, whereas the chain-type SCO structure yielded a NiO_6_ octahedron in LNO, allowing us to selectively control the out-of-plane and in-plane orbital occupancies. This type of oxygen coordination manipulation could enhance the Ni orbital polarization up to ~30%. This enhancement is larger than that achieved from other means, including by strain (~19%) or spatial confinement (~5%). Our findings unambiguously demonstrate how the interfacial oxygen coordination engineering can effectively manipulate the orbital configuration and electronic properties, providing a possible new approach to realizing superconducting nickelates with high *T*_c_. The oxygen coordination control using SCO as a template could also be applied to other perovskites to explore previously inaccessible orbital-driven phenomena.

## Methods

### Sample growth

All superlattices and thin films were grown on TiO_2_ terminated (001) SrTiO_3_ substrates by pulsed laser epitaxy using a KrF excimer laser (*λ* = 248 nm). The single TiO_2_ terminated and atomically flat SrTiO_3_ substrates were obtained by the etching with standard buffered-HF and subsequent annealing at 950 ^o^C for 1.5 h. The laser fluence and repetition rate were fixed at 2 J cm^−2^ and 5 Hz, respectively. The oxygen partial pressure and substrate temperature were maintained at 100–150 mTorr and 650 ^o^C, respectively.

### Sample characterization

The surface morphology was characterized by atomic force microscopy. The lattice structure was characterized by a high-resolution four-circle X-ray diffractometer. Transport measurements were performed in a van-der-Pauw geometry using a Physical Property Measurement System. The TEY and FY XAS measurements of the Cu and Ni edges were performed at the beamline 4–ID–C of the Advanced Photon Source. XAS was measured at 300 K, with the X-rays at a grazing (20°) angle to the films surface, and the TFY detector at 90° from the incoming X-rays.

## Supplementary information

Supplementary Information

## Data Availability

The data that support the findings of this study are available from the corresponding author upon reasonable request.

## References

[1] Tokura Y, Nagaosa N (2000). Orbital physics in transition-metal oxides. Science.

[2] Goodenough JB (1955). Theory of the role of covalence in the perovskite-type manganites [La,M(II)]MnO_3_. Phys. Rev..

[3] Goodenough JB (1958). An interpretation of the magnetic properties of the perovskite-type mixed crystals La_1−x_Sr_x_CoO_3−λ_. J. Phys. Chem. Solids.

[4] Kanamorim J (1959). Superexchange interaction and symmetry properties of electron orbitals. J. Phys. Chem. Solids.

[5] Tokura Y, Tomioka Y (1999). Colossal magnetoresistive manganites. J. Magn. Magn. Mater..

[6] Keimer B, Kivelson SA, Norman MR, Uchida S, Zaanen J (2015). From quantum matter to high-temperature superconductivity in copper oxides. Nature.

[7] Aetukuri NB (2013). Control of the metal–insulator transition in vanadium dioxide by modifying orbital occupancy. Nat. Phys..

[8] Hwang HY (2012). Emergent phenomena at oxide interfaces. Nat. Mater..

[9] Ohshima R (2017). Strong evidence for d-electron spin transport at room temperature at a LaAlO_3_/SrTiO_3_ interface. Nat. Mater..

[10] Matsuno J (2017). Interface-driven topological Hall effect in SrRuO_3_-SrIrO_3_ bilayer. Sci. Adv..

[11] Chakhalian J (2007). Orbital reconstruction and covalent bonding at an oxide interface. Science.

[12] Chakhalian J (2006). Magnetism at the interface between ferromagnetic and superconducting oxides. Nat. Phys..

[13] Okamoto S (2017). Charge Transfer in Iridate-Manganite Superlattices. Nano. Lett..

[14] Nichols J (2016). Emerging magnetism and anomalous Hall effect in iridate–manganite heterostructures. Nat. Commun..

[15] Lee HN, Christen HM, Chisholm MF, Rouleau CM, Lowndes DH (2005). Strong polarization enhancement in asymmetric three-component ferroelectric superlattices. Nature.

[16] Petrie JR (2016). Enhanced Bifunctional Oxygen Catalysis in Strained LaNiO3 Perovskites. J. Am. Chem. Soc..

[17] Benckiser E (2011). Orbital reflectometry of oxide heterostructures. Nat. Mater..

[18] Frano A (2013). Orbital control of noncollinear magnetic order in nickel oxide heterostructures. Phys. Rev. Lett..

[19] Disa AS (2015). Orbital engineering in symmetry-breaking polar heterostructures. Phys. Rev. Lett..

[20] Gibert M, Zubko P, Scherwitzl R, Íñiguez J, Triscone JM (2012). Exchange bias in LaNiO_3_-LaMnO_3_ superlattices. Nat. Mater..

[21] Grisolia MN (2016). Hybridization-controlled charge transfer and induced magnetism at correlated oxide interfaces. Nat. Phys..

[22] van Veenendaal M (2008). Anomalous ground states at the interface between two transition-metal compounds. Phys. Rev. B.

[23] Chaloupka J, Khaliullin G (2008). Orbital order and possible superconductivity in LaNiO_3_/LaMO_3_ superlattices. Phys. Rev. Lett..

[24] Hansmann P (2009). Turning a nickelate fermi surface into a cupratelike one through heterostructuring. Phys. Rev. Lett..

[25] Sawada H, Morikawa Y, Terakura K, Hamada N (1997). Jahn-Teller distortion and magnetic structures in LaMnO_3_. Phys. Rev. B.

[26] Wu M (2013). Strain and composition dependence of orbital polarization in nickel oxide superlattices. Phys. Rev. B.

[27] Freeland JW (2011). Orbital control in strained ultra-thin LaNiO_3_/LaAlO_3_ superlattices. Europhys. Lett..

[28] Disa AS, Walker FJ, Ismail-Beigi S, Ahn CH (2015). Research update: orbital polarization in LaNiO_3_-based heterostructures. APL Mater..

[29] Fabbris G (2016). Orbital engineering in nickelate heterostructures driven by anisotropic oxygen hybridization rather than orbital energy levels. Phys. Rev. Lett..

[30] Tung IC (2013). Connecting bulk symmetry and orbital polarization in strained RNiO_3_ ultrathin films. Phys. Rev. B.

[31] Teske CL, Mueller-Buschbaum H, Über Erdalkalimetall‐Oxocuprate. V (1970). Zur Kenntnis von Ca_2_CuO_3_ und SrCuO_2_. Z. Anorg. Allg. Chem..

[32] Norton DP (1994). Superconductivity in SrCuO_2_-BaCuO_2_ superlattices: formation of artificially layered superconducting materials. Science.

[33] Zhong ZC, Koster G, Kelly PJ (2012). Prediction of thickness limits of ideal polar ultrathin films. Phys. Rev. B.

[34] Harter JW (2015). Doping evolution and polar surface reconstruction of the infinite-layer cuprate Sr_1−x_La_x_CuO_2_. Phys. Rev. B.

[35] Samal D (2013). Experimental evidence for oxygen sublattice control in polar infinite layer SrCuO_2_. Phys. Rev. Lett..

[36] Hawthorn DG (2011). Resonant elastic soft x-ray scattering in oxygen-ordered YBa_2_Cu_3_O_6+δ_. Phys. Rev. B.

[37] van der Laan G (1994). Sum rules and fundamental spectra of magnetic X-Ray dichroism in crystal field symmetry. J. Phys. Soc. Jpn..

[38] Cao YW (2015). Engineered Mott ground state in a LaTiO_3_/LaNiO_3_ heterostructure. Nat. Commun..

[39] Chancey CC, O’Brien MCM (1993). The Jahn-Teller effect: An introduction and current review. Am. J. Phys..

[40] Zhang JJ (2017). Large orbital polarization in a metallic square-planar nickelate. Nat. Phys..

[41] Izumi F (1987). Crystal structure of the orthorhombic form of Ba_2_YCu_3_O_7-x_ at 42 K. Jpn. J. Appl. Phys..

[42] Chen CT (1992). Out-of-plane orbital characters of intrinsic and doped holes in La_2-x_Sr_x_CuO_4_. Phys. Rev. Lett..

[43] Guo EJ (2018). Oxygen diode formed in nickelate heterostructures by chemical potential mismatch. Adv. Mater..

[44] Liu J (2010). Effect of polar discontinuity on the growth of superlattices. Appl. Phys. Lett..

[45] Wrobel F (2018). Digital modulation of the nickel valence state in a cuprate-nickelate heterostructure. Phys. Rev. Mater..

[46] Scherwitzl R (2011). Metal-insulator transition in ultrathin LaNiO_3_ Films. Phys. Rev. Lett..

[47] Boris AV (2011). Dimensionality control of electronic phase transitions in nickel-oxide superlattices. Science.

[48] King PDC (2014). Atomic-scale control of competing electronic phases in ultrathin LaNiO_3_. Nat. Nanotechnol..

[49] Sakai E (2013). Gradual localization of Ni 3d states in LaNiO_3_ ultrathin films induced by dimensional crossover. Phys. Rev. B.

[50] Kumah DP (2014). Effect of surface termination on the electronic properties of LaNiO_3_ films. Phys. Rev. Appl..

[51] Kumah DP (2014). Tuning the structure of nickelates to achieve two-dimensional electron conduction. Adv. Mater..

