# Supplementary material for: Large orbital polarization in nickelate-cuprate heterostructures by dimensional control of oxygen coordination
Source: Nat Commun. 2019 Feb 4;10:589. doi: 10.1038/s41467-019-08472-y (PMC6362240; doi:10.1038/s41467-019-08472-y)
Supplement: Supplementary file 1 — Supplementary Information [file 41467_2019_8472_MOESM1_ESM.pdf]

Supplementary Information

Large orbital polarization in nickelate-cuprate heterostructures by dimensional control of oxygen coordination

Liao *et al.*

Supplementary Note 1. Epitaxial growth of $SrCuO_2$ and $LaNiO_3$ films

All the superlattices and thin films were grown on TiO_2 terminated (001) $SrTiO_3$ (STO) substrates by pulsed laser deposition using a 248-nm-wavelength KrF excimer laser. The STO substrates were treated by etching with buffered-HF (BHF) and subsequent annealing at >950 ^{o}C for 1.5 h.[1] A single unit step height (~ 0.4 nm) and atomically flat terrace were achieved for all STO substrates as confirmed by atomic force microscopy (AFM). High-quality, (001)-oriented infinite layer structure $SrCuO_2$ (SCO) was achieved as shown in Fig. 1, as one can find from the appearance of the thickness fringes (See Fig. 1b). With the same growth condition, high quality $LaNiO_3$ (LNO) films were obtained as well. The growth rates of SCO and LNO were calibrated from x-ray reflectivity.

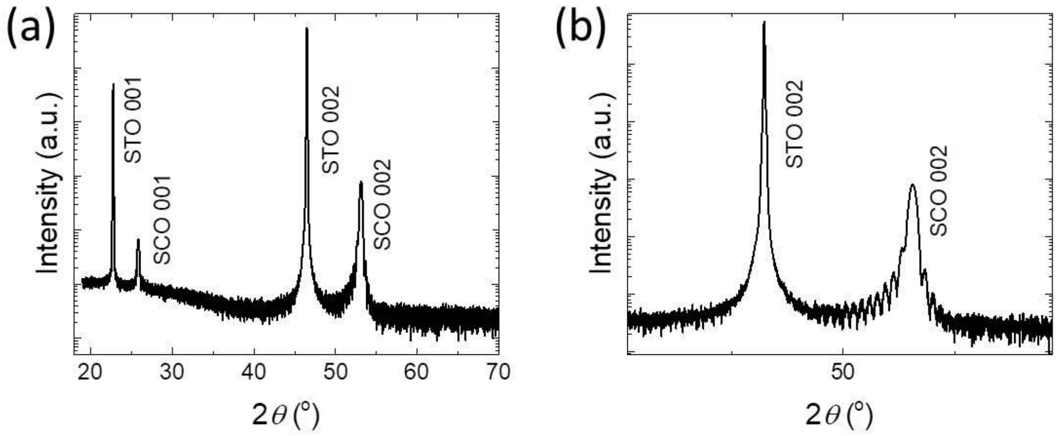

Supplementary Figure 1: Characterization of structure of $SrCuO_2$ films. (a) X-ray diffraction (XRD) θ-2θ scans of a $SrCuO_2$ (SCO) film (28 nm) grown on a $SrTiO_3$ (STO) substrate. A zoomed-in area around the 002 peak is shown in (b).

The $(SCO_n/LNO_m)_N$ (or $(S_n/L_m)_N$) superlattices were obtained by alternately stacking SCO and LNO layers. The thickness of individual layer was controlled by the number of the laser pulses according to the growth rate. Figure 2 shows the surface morphology of several different superlattices ($(S_7/L_1)_{15}$, $(S_3/L_1)_{25}$, $(S_7/L_4)_{15}$, $(S_3/L_4)_{15}$). All the films show atomically flat surfaces with clearly visible step-terrace features.

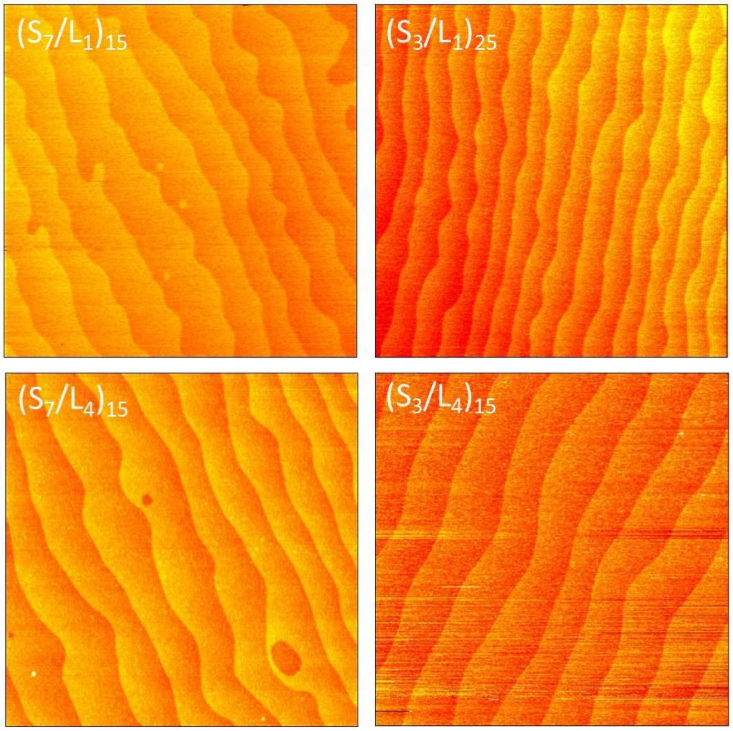

Supplementary Figure 2: Surface morphology of $SrCuO_2$-$LaNiO_3$ superlattices. Surface morphology of $(S_7/L_1)_{15}$, $(S_3/L_1)_{25}$, $(S_7/L_4)_{15}$ and $(S_3/L_4)_{15}$ superlattices characterized by atomic force microscopy (AFM), confirming our growth control for high-quality superlattice samples.

Supplementary Note 2. Thickness driven structural transformation in $SrCuO_2$

Besides the demonstrated structure transformation of SCO in S_N/L_1 superlattices in the main text, the reducing thickness driven transition from the planar-type structure to the chain-type structure in SCO occurred for different thicknesses of LNO within superlattices. Figure 3 shows θ-2θ scans of S_N/L_2 and S_N/L_3 superlattices. For S_3/L_2 and S_3/L_3, the superlattice main peaks SL(0) are located near 48°, which is close to the LNO 002 peak. XRD spectra of S_7/L_2 and S_7/L_3 superlattices are quite different from S_3/L_2 and S_3/L_3. The main peaks are located in between the SCO 002 and the LNO 002 peak. These results suggest a chain-type structure in S_3/L_N superlattices and planar-type structure in S_7/L_N superlattices.

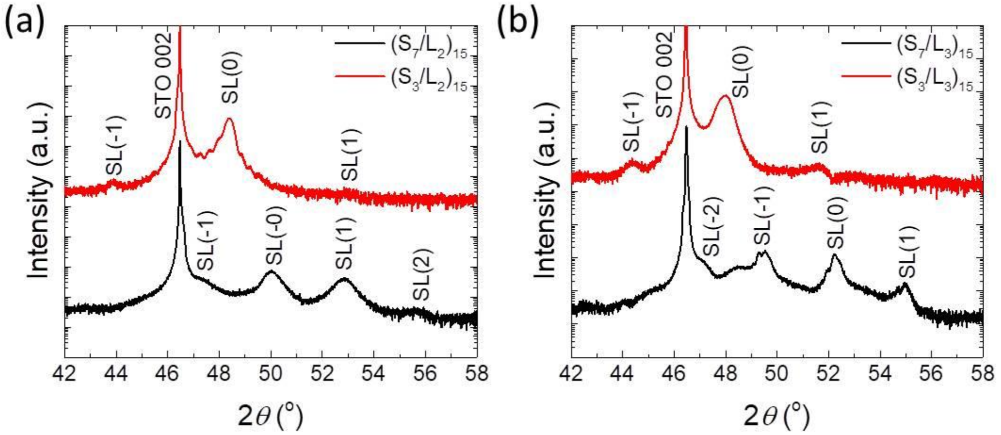

Supplementary Figure 3: **Structural characterization of $SrCuO_2$-$LaNiO_3$ superlattices**. X-ray diffraction (XRD) θ-2θ scans of (a) S_7/L_2 and S_3/L_2 superlattices; (b) S_7/L_3 and S_3/L_3 superlattices.

Supplementary Note 3. Characterization of domain structure in chain-type $SrCuO_2$

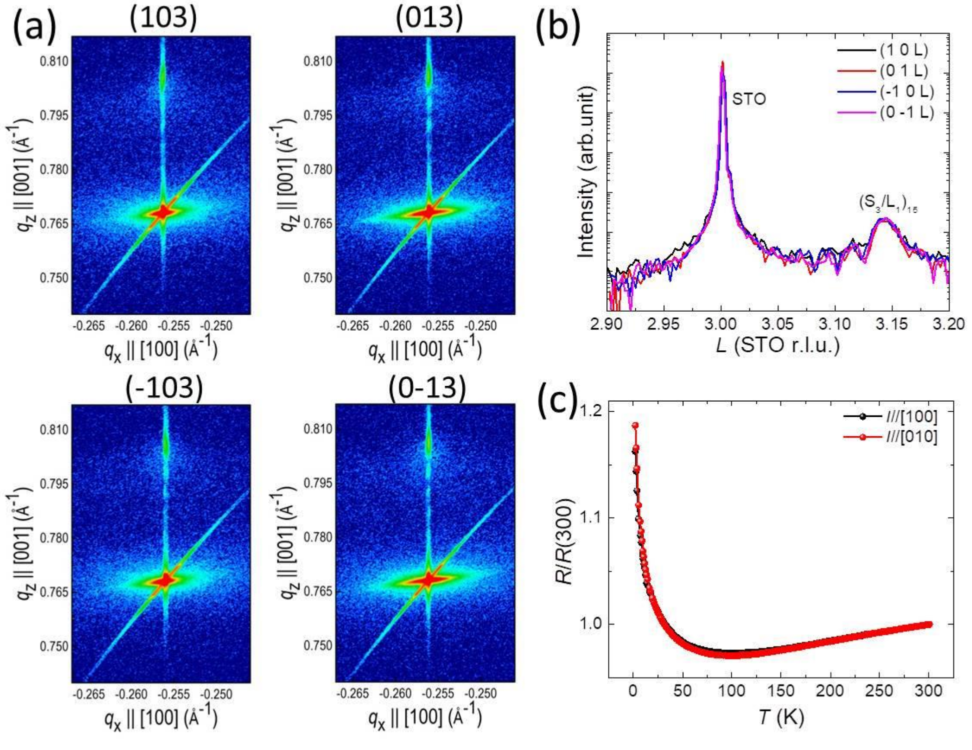

Supplementary Figure 4: **Characterization of domain structure in chain-type $SrCuO_2$.** (a) Reciprocal space mappings (RSMs) of around the (103), (013), (-103), (-013) peaks of $(SCO_3/LNO_1)_{15}$ superlattices. (b) *L*-scans along (00L) for (±1 0 L) and (0 ±1 L) peaks. (c)Temperature dependent resistance for current parallel to [100] (black curve) and [010] (red curve).

For a chain-type SCO, where the CuO_2 plane is lying along one of the in-plane principal axes, it has a two-fold in-plane symmetry. It is expected to see different peak intensities between (±103) and (0±13) peaks. Since the substrate $SrTiO_3$ (001) is cubic with a four-fold symmetry, the substrate should equivalently favor the *x*-*z* (CuO_2 plane parallel to the *x*-*z* plane) and *y*-*z* domains (CuO_2 plane parallel to the *y*-*z* plane). It is expected to see the formation of structural twinning. To investigate the domain structure, we performed reciprocal space mapping (RSM) of (103), (013) and (-103) and (0-13) peaks of the $(SCO_3/LNO_1)_{15}$ superlattices by rotating the sample by every 90 degree with respect to the surface normal.

Figure 4a shows RSMs around the (103), (013), (-103), (-013) peaks. All the superlattice peaks are aligned with the STO substrate peaks and are of a similar intensity. The direct comparison of the peak intensity is plotted in Fig. 4b, clearly demonstrating nearly identical superlattice peak intensities among the (103), (013), (-103), (-013) peaks. This result suggests a two-fold domain structure in the sample instead of a single-domain configuration.

The twin structures are also evident from the observed isotropic transport. There is a big different for electron hopping along the Cu-O-Cu chain from electron hopping from CuO_2 plane to another CuO_2 plane, resulting in anisotropic transport properties. However, the transport measurement shows isotropic conductivity for the two in-plane directions (see Fig. 4c).

Supplementary Note 4. X-ray linear dichroism of $SrCuO_2$ films

The in-plane (σ) and out-of-plane (π) polarized x-ray absorption spectroscopy (XAS) were used to probe $3d_{x^2-y^2}$ and $3d_{3z^2-r^2}$ orbital, respectively. Figure 5a shows XAS of Cu $L_{2,3}$-edge of a 28 nm-thick SCO film. Highly strong polarization dependent spectra were observed. The spectrum with x-ray polarized in-plane ($I(\sigma)$) has a much higher intensity than that with out-of-plane polarized x-ray ($I(\pi)$), suggesting that most of the holes occupy $3d_{x^2-y^2}$ orbital, well consistent with typical layer structure (see Fig. 5b) induced orbital configuration in cuprate (see Fig. 5c).

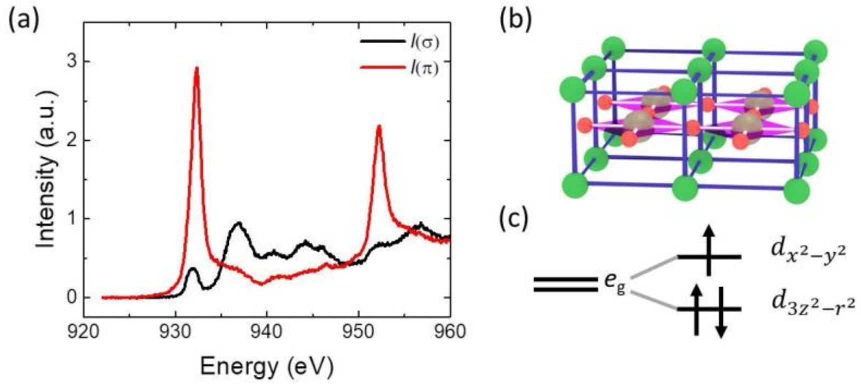

Supplementary Figure 5: Orbital configuration of planar-type $SrCuO_2$ (SCO). (a) Polarization dependent x-ray absorption spectroscopy for a 28 nm-thick SCO film grown on a $SrTiO_3$ substrate. (b) and (c) show the lattice structure and e_g orbital energy splitting of SCO, respectively.

Supplementary Note 5. Microscopic investigation of structure transformation

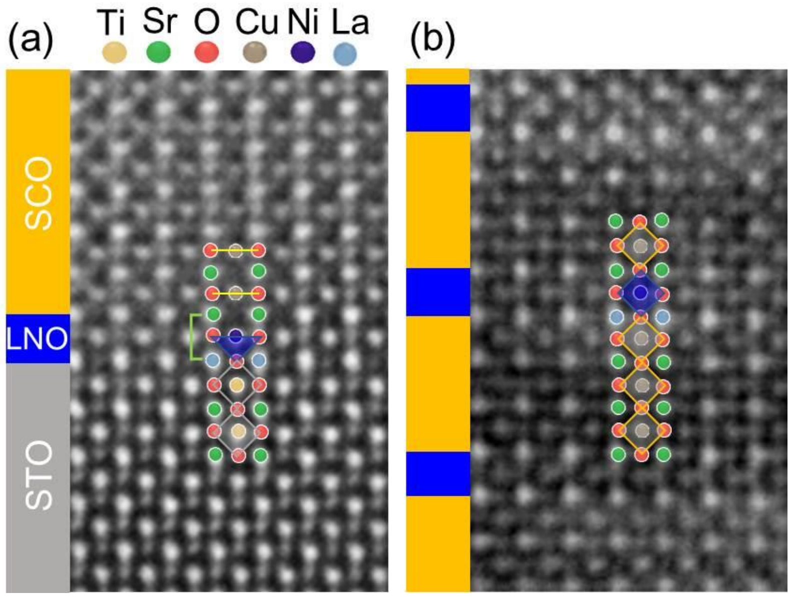

Supplementary Figure 6: Microscopic investigation of interfacial oxygen coordination. Contrast inverted annular bright field images (I-ABF) of (a) S_7/L_1 and (b) S_3/L_1 superlattices.

The reduced thickness driven planar-type SCO to chain-type SCO transformation and accordingly change of oxygen coordination of Ni were further investigated by scanning transmission electron microscopy (STEM). Figure 6 shows the contrast inverted annular

bright field (ABF) images of S_7/L_1 and S_3/L_1 superlattices. Inverted contrast is used to easier distinguish the atom positions.[2] In the S_7/L_1 superlattice, where SCO is expected to be planar-type structure suggested from XRD and linearly polarized x-ray absorption spectroscopy shown in main text, we observed clearly the missing of apical oxygen in SCO layer. This in term results in pyramid NiO_5 structure at interface (see Fig. 6a). In contrast, the apical oxygen is seen in S_3/L_1 superlattice and NiO_6 octahedral structure is formed at interface (see Fig. 6b). Our results prove the change of oxygen coordination microscopically.

Supplementary Note 6. Polarized x-ray absorption spectroscopy of Ni $L_{2,3}$-edge

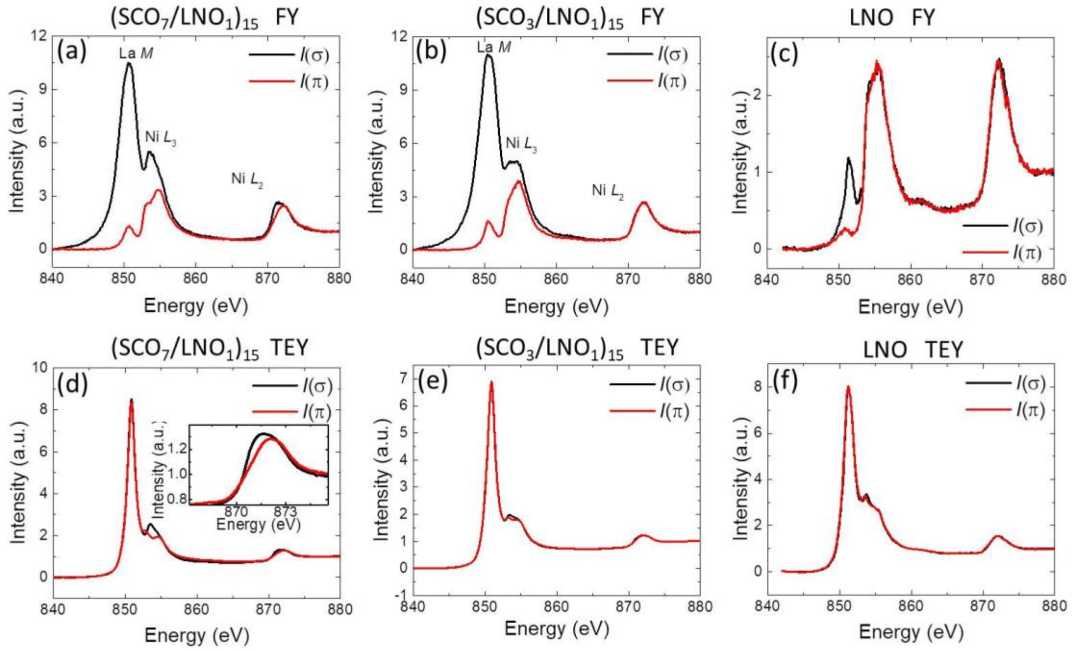

Supplementary Figure 7: Comparison of fluorescence yield mode and total electron yield mode. Polarization dependent x-ray absorption spectroscopy of Ni $L_{2,3}$-edge in FY mode (a-c) and TEY mode (d-f) for samples (a) and (d) $(SCO_7/LNO_1)_{15}$, (b) and (e) $(SCO_3/LNO_1)_{15}$, (c) and (f) LNO.

The full spectra of Ni $L_{2,3}$-edges are shown in Fig. 7. For both S_7/L_1 and S_3/L_1 superlattices and 34 nm LNO film, strong polarization dependent XAS of La M-edge was observed in fluorescence yield (FY) mode but not in total electron yield (TEY) mode. Such huge polarization in FY is due to localized $4f^0$ electron of La which makes the FY spectrum deviated from true XAS.[3] The polarized x-rays excite the 3d core electron

into a specific empty 4*f* orbital. Given that incoming x-rays come in along the *x* axis, and fluorescence detector is on the *z* axis. In this case, π-polarized x-rays would be polarized in the *z*-direction, and this would excite the 3d core electron into a specific 4*f* orbital. Since La is $4f^0$, the electron that decays back to the 3d orbital has to be the same electron that is excited to 4*f* orbital. As a result, the radiation that is given off has to be *z*-polarized as well. But if the detector is on the *z*-axis at 90 degrees from the incident beam, it can't detect the decay process, because it can only detect *x*- and *y*-polarized photons coming from the sample (polarization has to be orthogonal to poynting vector). If instead the incident x-rays are σ-polarized, this would be polarized in the *y*-direction. The outgoing fluorescence photon would also be polarized in the *y*-direction, and hence can be detected by fluorescence detector on the *z*-axis. Such selection rule intrinsically induces a huge polarization in La $M_{4,5}$ edge. The strong overlap of Ni L_3-edge with La *M*-edge leads to a large linear dichroism in Ni L_3-edge in FY. This is more obvious in S_3/L_1 superlattice. There is no linear dichroism in Ni L_2-edge ($I(\sigma) = I(\pi)$), but strong linear dichroism in Ni L_3-edge.

Supplementary Note 7. Quantitative evaluation of the orbital polarization

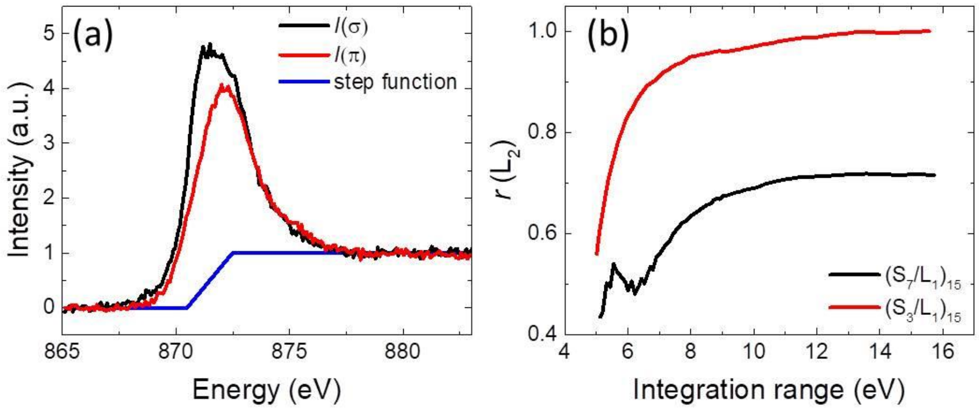

Supplementary Figure 8: **Hole ratio evaluation method.** (a) X-ray absorption spectroscopy (XAS) of a S_7/L_1 superlattice and the step function used for subtracting the background. (b) The hole ratio *r* as a function of integration window.

Since the Ni L_3-edge strongly overlaps with La *M*-edge, we are not able to directly integrate full $L_{2,3}$-edge intensity in order to use the sum rule to quantify the x^2-y^2 to $3z^2$-r^2

ratio. However, Zhang *et al.* illustrated that the sum rule for L_2-edge in the dipole approximation leads to the identical formula as shown in the main text.[4]

$$r = \frac{h_{3z^2-r^2}}{h_{x^2-y^2}} = \frac{3I(\mathrm{c})}{4I(\mathrm{ab})-I(\mathrm{c})} \qquad (1)$$

Therefore, we can still use the above formula to evaluate the orbital polarization. Similar to the background treatment by Zhang *et al.*,[4] a step function background was subtracted to remove excitations into continuum (see Fig. 8a) and then we integrated the peak (I(ab), I(c)) intensity. Since there is an angle $\theta = 20^{\circ}$ between incident x-ray and the sample surface in our experiment setup, the $I(\pi)$ needs to be corrected and $I(\mathrm{c})=(I(\pi)-I(\mathrm{ab})\mathrm{Sin}^2\theta)/\mathrm{Cos}^2\theta$. Figure 8b shows results of r as a function of integration range with starting point at 865 eV. The r converges at about 12 eV integration range. It is found that $r(S_7/L_1)$ and $r(S_3/L_1)$ are 0.7 and 1.0, respectively.

Supplementary Note 8. X-ray absorption spectroscopy of Ni *L*-edge in $LaNiO_3$ film.

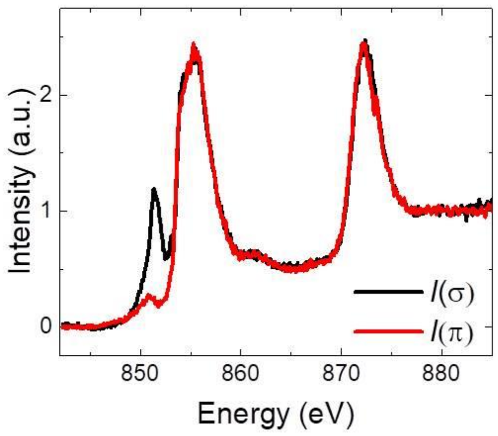

Supplementary Figure 9: **Orbital polarization of $LaNiO_3$ film.** Polarization dependence of the x-ray absorption spectroscopy of Ni $L_{2,3}$-edge of a $LaNiO_3$ film (34 nm) grown on a $SrTiO_3$ substrate.

X-ray polarization dependent XAS of Ni L_2-edge of a 34 nm-thick LNO film on a STO substrate is shown in Fig. 9. The σ- and π-polarized XAS of Ni $L_{2,3}$-edges share the same intensity. Therefore, there is no orbital polarization although the LNO film is fully strained to the STO and therefore has large tensile strain. This result is consistent with previous report by Tung *et al.*.[5]

Supplementary Note 9. Transport properties of $SrTiO_3$-$LaNiO_3$ superlattices

The transport properties of $(STO_7/L_N)_{15}$ superlattices is shown in Fig. 10. With reducing the thickness (N) of LNO sublayer, the superlattices exhibit a metal to insulator transition. Superlattices with thickness of 2 u.c. or below (i.e., N = 1, 2 u.c.), the superlattices are insulating, but above 2 u.c., the superlattices are metallic. Therefore, the critical thickness for MIT in $(STO_7/L_N)_{15}$ is 2 u.c. The upturn of resistance at low temperature observed in 3 and 4 u.c. LNO and is due to the weak localization effect.[6]

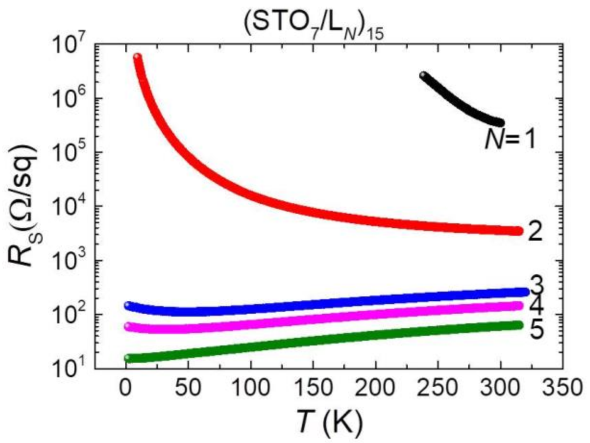

Supplementary Figure 10: Metal to insulator transition in $((SrTiO_3)_7/(LaNiO_3)_N)_{15}$ superlattices. Temperature dependent sheet resistance of $((SrTiO_3)_7/(LaNiO_3)_N)_{15}$ superlattices with varying LNO sublayer thickness (N) from 1 to 5 u.c.

Supplementary References:

[1] G. Koster, B. L. Kropman, G. J. H. M. Rijnders, D. H. A. Blank, and H. Rogalla, Quasi-ideal strontium titanate crystal surfaces through formation of strontium hydroxide, *Appl. Phys. Lett.***73**, 2920 (1998).

[2] Z. L. Liao, M. Huijben, Z. Zhong, N. Gauquelin, S. Macke, R.J. Green, S. Van Aert, J. Verbeeck, G. Van Tendeloo, K. Held, G.A. Sawatzky, G. Koster, G. Rijnders, Controlled lateral anisotropy in correlated manganite heterostructures by interface-engineered oxygen octahedral coupling, *Nat. Mater.* **15**, 425 (2016).

[3] R. J. Green, D. Peak, A. J. Achkar, J. S. Tse, A. Moewes, D. G. Hawthorn, and T. Z. Regier, Comment on "State-Dependent Electron Delocalization Dynamics at the Solute-Solvent Interface: Soft-X-Ray Absorption Spectroscopy and Ab initio Calculations", *Phys. Rev. Lett.* **112**, 129301 (2014).

[4] J. J. Zhang, , A. S. Botana, J. W., Freeland, D. Phelan, H. Zheng, V. Pardo, M. R. Norman and J. F. Mitchell, Large orbital polarization in a metallic square-planar nickelate, *Nat. Phys.* **13**, 864 (2017).

[5] I. C. Tung, P. V. Balachandran, J. Liu, B. A. Gray, E. A. Karapetrova, J. H. Lee, J. Chakhalian, M. J. Bedzyk, J. M. Rondinelli and J. W. Freeland, Connecting bulk symmetry and orbital polarization in strained $RNiO_3$ ultrathin films, *Phys. Rev. B* **88**, 205112 (2013).

[6] R. Scherwitzl, S. Gariglio, M. Gabay, P. Zubko, M. Gibert, M. and J.-M.Triscone, Metal-insulator transition in ultrathin $LaNiO_3$ Films, *Phys. Rev. Lett.* **106**, 246403 (2011).
